# Evaluation of aromatic amino acids as potential biomarkers in breast cancer by Raman spectroscopy analysis

**DOI:** 10.1038/s41598-021-81296-3

**Published:** 2021-01-18

**Authors:** Shaymus Contorno, Richard E. Darienzo, Rina Tannenbaum

**Affiliations:** 1 Department of Materials Science and Chemical Engineering, Stony Brook University, Stony Brook, NY 11794 USA; 2 The Stony Brook Cancer Center, Stony Brook University, Stony Brook, NY 11794 USA

**Keywords:** Cancer, Breast cancer, Cancer imaging, Cancer screening, Raman spectroscopy

## Abstract

The scope of the work undertaken in this paper was to explore the feasibility and reliability of using the Raman signature of aromatic amino acids as a marker in the detection of the presence of breast cancer and perhaps, even the prediction of cancer development in very early stages of cancer onset. To be able to assess this hypothesis, we collected most recent and relevant literature in which Raman spectroscopy was used as an analytical tool in the evaluation of breast cell lines and breast tissue, re-analyzed all the Raman spectra, and extracted all spectral bands from each spectrum that were indicative of aromatic amino acids. The criteria for the consideration of the various papers for this study, and hence, the inclusion of the data that they contained were two-fold: (1) The papers had to focus on the characterization of breast tissue with Raman spectroscopy, and (2) the spectra provided within these papers included the spectral range of 500–1200 cm^−1^, which constitutes the characteristic region for aromatic amino acid vibrational modes. After all the papers that satisfied these criteria were collected, the relevant spectra from each paper were extracted, processed, normalized. All data were then plotted without bias in order to decide whether there is a pattern that can shed light on a possible diagnostic classification. Remarkably, we have been able to demonstrate that cancerous breast tissues and cells decidedly exhibit overexpression of aromatic amino acids and that the difference between the extent of their presence in cancerous cells and healthy cells is overwhelming. On the basis of this analysis, we conclude that it is possible to use the signature Raman bands of aromatic amino acids as a biomarker for the detection, evaluation and diagnosis of breast cancer.

## Introduction

**Figure 1:**
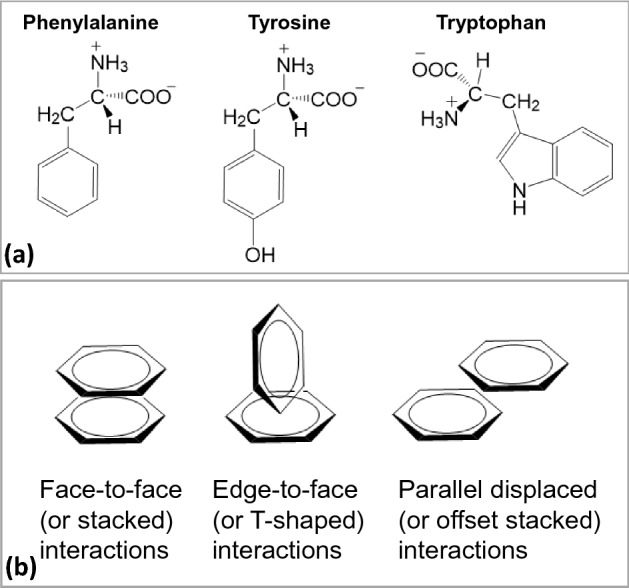
(**a**) The chemical structure of the three phenyl-based aromatic amino acids phenylalanine, tyrosine and tryptophan, and (**b**) The schematic representation of the three types of interactions between the phenyl rings of the aromatic amino acids.

Phenylalanine, tyrosine and tryptophan, shown in Fig. 1a, are the only three amino acids that contain benzyl-based aromatic groups, and hence, are referred to as aromatic amino acids (AAA). These residues have a rigid, planar structure and possess added stability due to the π-electron cloud situated above and below the plane of the aromatic ring^1–3^. The AAA residues can undergo aromatic-aromatic interactions, consisting of hydrogen bonding coupled with attractive, noncovalent, dipole and van der Walls interactions and pi-stacking of the benzene rings^4–8^. These interactions result in an optimal geometry where the partially-charged positive hydrogens of the C–C edges in one ring interact favorably with the π-electrons and partially-charged negative carbons of the other ring. The π-system of the aromatic rings gives rise to three types of interactions involving aromatic moieties: (a) π–π alignment of the aromatic residues, (b) Dipole-π system attraction of cations within 6.0 Å of the face of an aromatic ring, and (c) Long range CH-π interactions^6^. These interactions give rise to three different types of geometries involving two aromatic residues, as shown in Fig. 1b: (a) Edge-to-face (or T-shaped), (b) Face-to-face (or stacked)^7^, and (c) Parallel displaced (or offset stacked) interactions. These aromatic-aromatic interactions have been found to play an important role in the stabilization of the overall structure of protein molecules^9–14^ and protein-DNA/RNA complexes through free energy lowering by − 0.6 and − 1.3 kcal/mol, respectively^15–17^. Hence, the interactions between the aromatic residues within a protein and in protein-DNA/RNA complexes constitute a crucial element in the proper functioning of protein molecules, which in turn impacts various cellular processes in which these proteins take part^5,18^.

Besides the structural function in proteins, aromatic amino acids are precursors to many biological compounds that are essential for the normal functioning of live organisms^18,19^. Many of these compounds may be involved in the pathogenesis of many diseases, such as cancers, psychiatric disorders, depression, cardiovascular diseases. chronic kidney insufficiency or diabetes. In recent years, the impact of the variations in aromatic amino acid content and sequence has been the focus of much effort in understanding the nature of disordered proteins and their involvement in cancer pathology, as well as the impact of proper membrane functioning. It is well established that metabolic abnormalities occur prior to significant morphological changes in malignant tissue^20^. For example, high levels of aromatic amino acids that are present in gastric juice have been deemed indicative of the onset of early gastric cancer (ECG)^21^. Since gastric juice characteristics contain considerable information concerning the metabolic state of the gastric epithelium, high levels of aromatic amino acids could constitute valid biomarkers for the detection of such a cancer^21,22^.

Increased concentrations of AAA have been found in additional various cancer types, such as prostate, lung, breast, oral and esophageal tissue samples. For example, it is well known that some tryptophan metabolites exhibit active involvement in carcinogenesis. Indolic derivatives of tryptophan have been shown to activate the aryl hydrocarbon receptor, which in turn can be transported to the nucleus and bind to a specific DNA sequence, thus acting as a transcription factor. Hence, tryptophan metabolites that are not carcinogens by themselves can promote a neoplastic process when coupled with other enzymatic reactions^23–26^. This has been shown to occur in urinary bladder cancer and lung cancer. The tryptophan metabolites, in addition to acting as chimeric transcription factors, are also involved in the development of tumor-induced immunosupression through proliferation inhibition and promotion of lymphocyte-T apoptosis, as is the case in some types of breast cancer. In addition to tryptophan, also tyrosine and phenylalanine can constitute the precursors to wide array of substances that may have mutagenic, genotoxic and carcinogenic properties, such as phenolic and indolic compounds^25,27,28^. The overexpression of these molecules in colon tissue may be indicative of the onset of colon cancer.

The enhanced expression of aromatic amino acids in breast cancer became apparent in a multitude of recent studies, in which cancer tissue characteristics was measured by Raman spectroscopy^29–49^. The SK-BR-3 cell lines, characterized by the overexpression of HER-2 receptor, and MDA-MB-231 cell lines, characterized by the lack of estrogen, progesterone and HER-2 receptors, exhibited intense Raman bands that correspond to aromatic amino acids such as tyrosine, phenylalanine, and tryptophan^29^, particularly when using the surface enhance Raman spectroscopy technique. Raman spectroscopy has emerged in recent years as a successful tool for the diagnosis of breast cancer tissue samples by detecting lipid and protein identity, structure and concentrations based on known samples or difference spectra. If the spectral profiles acquired via Raman spectroscopy possess high enough intensity and signal-to-noise ratios, then the subcellular changes present in different cancer phenotypes should be discernible. In view of the considerable involvement and enhanced presence of aromatic amino acids in malignant tissues, they could also be used as an important marker in the diagnosis and prediction of cancer onset.

The scope of the work undertaken in this paper was to explore the feasibility and reliability of using the Raman signature of aromatic amino acids as a marker in the detection of the presence of breast cancer and perhaps, even the prediction of cancer development in very early stages of cancer onset. To be able to assess this hypothesis, we collected most recent and relevant literature in which Raman spectroscopy was used as an analytical tool in the evaluation of breast cell lines and breast tissue, re-analyzed all the Raman spectra, and extracted all spectral bands from each spectrum that were indicative of aromatic amino acids. All data were then plotted without bias in order to observe whether there is a pattern that can shed light on a possible diagnostic classification.

## Results and discussion

**Figure 2:**
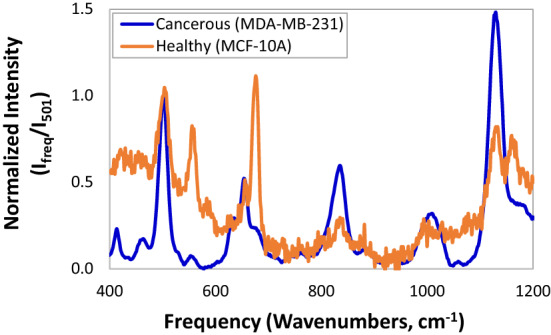
Typical Raman spectra of healthy breast cells and cancerous breast cells. The cancerous cells belong to the MDA-MB-231 cell line and consist of cancer cells that lack estrogen receptors (ER), progesterone receptors (PR), and HER2 amplification, and are also known as "triple negative". The healthy cells belong to the MCF-10A cell line and are benign cells with fibrocystic changes. The spectra were normalized with respect to the intensity of the 501 cm^−1^ peak belonging to the underlying Si substrate onto which the cell samples were placed.

Typical Raman spectra of healthy breast cells and cancerous breast cells are shown in Fig. 2. The two human-mammary epithelial cell lines consist of the MCF-10A cells, which are benign cells with fibrocystic changes and MDA-MB-231 cells, which are cancer cells that lack estrogen receptors (ER), progesterone receptors (PR), and HER2 amplification, and are also known as “triple negative”. The spectra were normalized with respect to the intensity of the 501 cm^−1^ peak belonging to the underlying Si substrate onto which the cell samples were placed.

**Figure 3:**
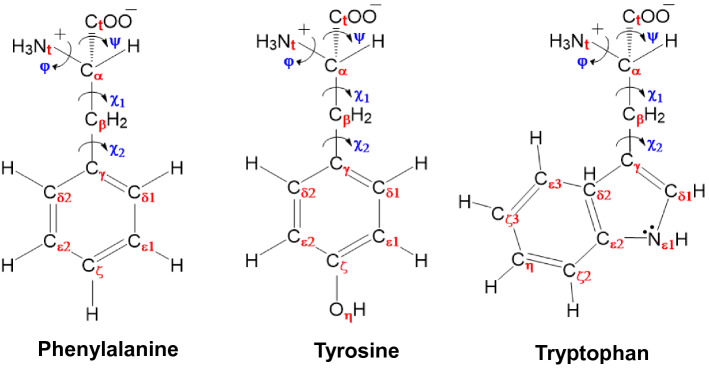
The assigned atom identities and main conformational angles of the zwitterionic form of the aromatic amino acids phenylalanine, tyrosine, and tryptophan. Nt and Ct refer to the main atoms involved in the terminal charged groups, φ and ψ are the backbone conformational angles that are represented by (Ct–Cα–Nt–H) and (Nt–Cα–Ct–O), respectively. χ1 and χ2 are the side chain conformational torsion angles that are represented by (Nt–Cα–Cβ–Cγ) and (Cα–Cβ–Cγ–Cδ1), respectively. Note that for phenylalanine, the phenyl ring carbon atoms C*δ*1 and C*δ*2 are equivalent.

The Raman shift peak frequencies that we followed and analyzed correspond to those characteristic of aromatic amino acids at 657, 816, 837, 845, 965, 1006, 1012 and 1131 cm^–1^^29,54–58^, and to S–S bonds at 503 cm^−1^^29^. The assignment of the vibrational modes of these peaks is shown in Fig. 3 and summarized in Table 1.

**Table 1:** Summary of the Raman vibrational peaks and the assignment of the vibrational modes of the three main aromatic amino acids: phenylalanine, tyrosine and tryptophan.

Wavenumber (cm^−1^)	Molecular origin	Vibrational mode assignment	References
503	S–S bonds	Vibration if S–S disulfide bridge	^29^
657	Tyrosine	In plane ring vibration (6B) Cγ–Cδ1–Cε1, Cγ–Cδ2–Cε2, Cδ1–Cε1–Cζ, Cζ–Cε2–Cδ2	^54–58^
816	Phenylalanine	Cβ–Cγ; Cγ–Cδ1, Cε1–Cζ–Cε2	^56,58^
837	Tyrosine	Out of plane bending (Cε2–H) and (Cζ–O)	^56,58^
845	Tyrosine	Out of plane bending (Cε2–H)	^56,58^
965	Tyrosine	Out of plane ring vibration (17a) Cα–Cβ and Water(H)···O(Ct)	^54–58^
1006	Phenylalanine	Phenyl ring angular bending Cγ–Cδ1, Cγ–Cδ2, Cδ2–Cγ–Cε1, Cγ–Cδ2–Cε2, Cζ–Cε1–Cδ1	^56,58^
1012	Tryptophan	Symmetric benzene/pyrrole out of phase breathing mode Cζ3–Cη, Cζ2–Cη, Cε3–Cζ3	^56,58^
1131	Tryptophan	NtH_3_^+^ asymmetric rock and Cη–Cζ2–H	^56,58^

Phenylalanine has two main characteristic bands: one is at 816 cm^−1^, corresponding to the in-plane vibrations between the substituted carbon and the immediately-bonded carbon of the side chain (Cγ–Cβ) and the double bond between the substituted carbon and its ortho neighbor (Cγ–Cδ1), and the cooperative vibration of the three ring carbons opposite the substituted carbon, (Cε1–Cζ–Cε3). The second is at 1006 cm^−1^, corresponding to the phenyl ring angular bending (i.e. distortion) due to contributions from the changes in the relative position between the substituted carbon and both its ortho neighbors (Cγ–Cδ1 and Cγ–Cδ2) and contributions from the cooperative vibrations of the conjugated double bond groups (Cδ2–Cγ–Cε1, Cγ–Cδ2–Cε2 and Cζ–Cε1–Cδ1). Tyrosine has a para-substituted phenyl ring, with a hydroxy group at the Cζ carbon. While the molecule is similar to phenylalanine, it has, however, a lower degree of symmetry due to the para-substitution and hence, exhibits 4 main characteristic bands. The band at 657 cm^−1^ corresponds to the in-plane ring vibration of the conjugated molecular fragments of the phenyl ring (Cγ–Cδ1–Cε1, Cγ–Cδ2–Cε2, Cδ1–Cε1–Cζ and Cζ–Cε2–Cδ2). The doublet bands at 837 and 845 cm^−1^ correspond to the out-of-plane bending along the bond between the unsubstituted phenyl ring carbons and associated hydrogen (e.g. Cε1–H and Cε2–H) and the out-of-plane bending along the carbon–oxygen bond in the hydroxyl group (Cζ–O). The fourth characteristic band at 965 cm^−1^ corresponds to the out-of-plane ring vibration of the two main carbons of the substituent functional group on the phenyl carbon (Cα–Cβ) and the hydrogen bonding associated with the carboxylic group on the terminal carbon and water (HO–H···OOCt–). Finally, tryptophan exhibits 2 characteristic band: one band is at 1012 cm^−1^, corresponding the symmetric benzene and/or pyrrole out of phase breathing modes (e.g. the cooperative vibrations of Cζ3–Cη, Cζ2–Cη and Cε3–Cζ3, and the second band is at 1131 cm^−1^, corresponding to the asymmetric rocking vibration of the terminal amine group (NtH_3_^+^) and the cooperative vibration modes of unsubstituted ring carbons and associated hydrogens (e.g. Cη–Cζ2–H). It is important to note that while the 1012 cm^−1^ band is characteristic of tryptophan, its intensity in all the spectra that were examined is very low and hence, has not been used in the overall peak analysis. Also, the S–S vibration peaks at 503 cm^−1^ are very weak and appear in all spectra, they are not specific to the aromatic amino acids, but rather ubiquitous in proteins as part of their tertiary structure, and therefore, were not included in the statistical analysis.

**Figure 4:**
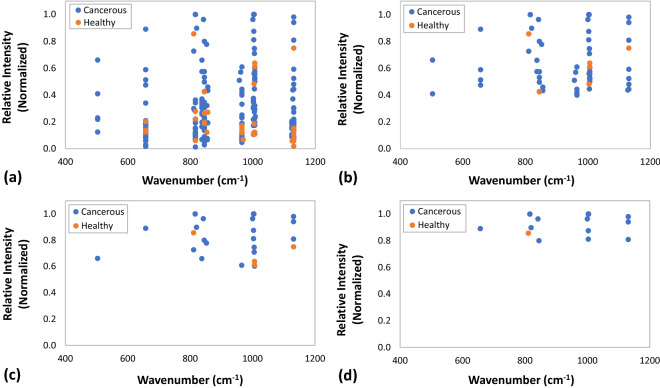
The collection of all the Raman bands in the spectral range of 500–1200 cm^−1^ that were extracted from the pertinent Raman spectra of breast tissues in the literature. (**a**) The collection of all the normalized Raman band intensities, (**b**) The normalized peaks having intensities in the upper 60th percentile (intensities ≥ 40%), (**c**) The normalized peaks having intensities in the upper 40th percentile (intensities ≥ 60%), and (**d**) The normalized peaks having intensities in the upper 20th percentile (intensities ≥ 80%). All the data points were classified into two cell categories, cancerous and healthy.

**Figure 5:**
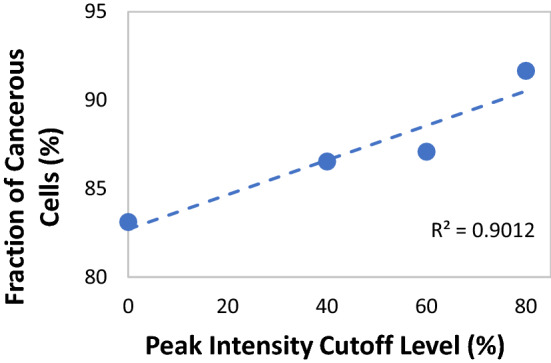
The fraction of the number of data points of aromatic amino acids originating from cancerous cells as a function of the cutoff for the relative intensity of the corresponding Raman bands. As the total number of points decreases as a function of the increase in the normalized intensity threshold, the statistical analysis of the contributions of both healthy and cancerous cells becomes less accurate. However, the trend is indicative of the fact that the characteristic Raman bands of aromatic amino acids in cancerous cells exhibited higher intensities and hence, were present in higher concentrations.

The collection of all the normalized Raman band intensities in the spectral range of 500–1200 cm^−1^ for all breast tissues examined^29–49^ are shown in Fig. 4a. All the peaks collected from the pertinent Raman spectra in the literature were grouped into two cell categories, cancerous and healthy, the latter category including also cells that have been deemed benign (i.e. non-cancerous) in their respective publications^29–49^. It is evident from this representation of the data, that at higher relative peak intensities, most of the peaks belong to the cancerous cells across all frequencies. The statistical analysis indicates that most of the data points assigned to aromatic amino acids in the analyzed spectra represent Raman bands generated by cancerous cells (or tissues) as they comprise 83% of the data, while healthy cells comprise only over 16%. In order to better highlight this trend, we replotted the normalized peaks having intensities in the upper 60th percentile (intensities ≥ 40%), shown in Fig. 4b, the upper 40th percentile (intensities ≥ 60%), shown in Fig. 4c and in the upper 20th percentile (intensities ≥ 80%), shown in Fig. 4d. As the cutoff for the relative intensity increased, so did the fraction of the data points of aromatic amino acids originating from cancerous cells, as shown in Fig. 5. While the statistical analysis of the contributions of both healthy and cancerous cells becomes less accurate as the total number of data points decrease at higher relative intensity thresholds, it nevertheless, indicates that the characteristic Raman bands of aromatic amino acids in cancerous cells exhibit higher intensities and hence, they are present in higher concentrations^33,43,45^. This conclusion is in accordance with various observations regarding the enhanced prevalence of aromatic amino acids in cancerous tissues. For example, a comparison between the non-malignant MCF-10A with malignant SK-BR-3 breast cell lines indicates an increase in the concentration of tyrosine-rich proteins, as evidenced by the high intensity band at 837 cm^−1^, in accordance with the structure of the HER-2 receptor^29,59–62^. In addition, it has been observed that the Raman spectra corresponding to aromatic amino acids in malignant SK-BR-3 and MDA-MB-231 breast cell lines exhibit intense bands characteristic to tyrosine, phenylalanine, and tryptophan, suggesting the enhancement in the expression of such aromatic amino acid rich proteins in malignant cells^29,63–68^. This phenomenon is not restricted to breast cancer alone, but is prevalent in other malignancies such as pancreatic cancer, colon cancer, gastric cancer, and lung cancer. The overexpression of aromatic amino acids in proteins is considered as a factor that contributes to the destabilization of their tertiary structure and hence, induces the reduction in their 3D structural rigidity and the enhancement of protein disorder, a characteristic that is prevalent in oncoproteins involved in malignant tumor genesis and robustness^66,67^.

**Figure 6:**
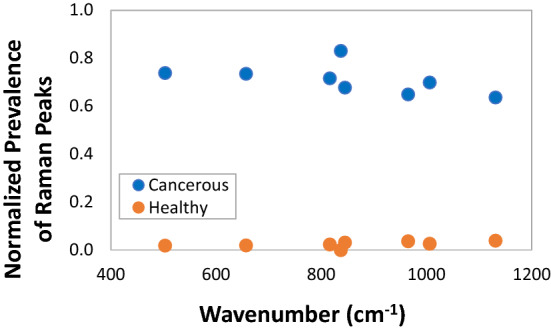
The statistical analysis of the normalized prevalence of the data points corresponding to aromatic amino acids in the analyzed Raman spectra for each cell type category. The normalization of data point abundance at a given Raman shift frequency was obtained by the fraction of data points at a particular frequency for one of the two cell categories multiplied by the weight of the particular cell category in the overall data population.

To further highlight the specificity of the enhanced presence of aromatic amino acids in cancerous cells, the normalized abundance of the data points corresponding to aromatic amino acids in the analyzed Raman spectra were grouped as a function of presence or absence of cell malignancy and of Raman shift frequency, and shown in Fig. 6. The normalization of data point abundance at a given Raman shift frequency was obtained by dividing the number of data points at a particular frequency for one of the two cell categories by the total number of data points at that frequency and the result was then further multiplied by the relative weight of the specific cell category in the overall data point population, as shown in Eq. (1):

1 $$ A_{{\nu_{i} }}^{{C_{1} }} = \frac{{P_{{\nu_{i} }}^{{C_{1} }} }}{{\left( {P_{{\nu_{i} }}^{{C_{1} }} + P_{{\nu_{i} }}^{{C_{2} }} } \right)}} \cdot \frac{{\sum\limits_{i} {P_{{\nu_{i} }}^{{C_{1} }} } }}{{\sum\limits_{i} {\left( {P_{{\nu_{i} }}^{{C_{1} }} + P_{{\nu_{i} }}^{{C_{2} }} } \right)} }}{\text{ and }}A_{{\nu_{i} }}^{{C_{2} }} = \frac{{P_{{\nu_{i} }}^{{C_{2} }} }}{{\left( {P_{{\nu_{i} }}^{{C_{1} }} + P_{{\nu_{i} }}^{{C_{2} }} } \right)}} \cdot \frac{{\sum\limits_{i} {P_{{\nu_{i} }}^{{C_{2} }} } }}{{\sum\limits_{i} {\left( {P_{{\nu_{i} }}^{{C_{1} }} + P_{{\nu_{i} }}^{{C_{2} }} } \right)} }} $$

where $A_{{\nu_{i} }}^{{C_{1} }}$ and $A_{{\nu_{i} }}^{{C_{2} }}$ are the normalized abundances of both cell categories, C_1_ and C_2_, at a specific Raman shift frequency ν_i_ and $P_{{\nu_{i} }}^{{C_{1} }}$ and $P_{{\nu_{i} }}^{{C_{2} }}$ are the number of data points for each cell category at that given frequency. The pattern that emerges shows that there is a distinct separation between the data points belonging to aromatic amino acids in healthy cells and those belonging to cancerous cells, with the latter occupying the more abundant regions of the plot. This indicates that the prevalence of aromatic amino acids in cancerous cells is more pronounced and there is a likelihood that this prevalence could be used as a diagnostic tool in identifying breast malignancy.

## Conclusions

The applicability of Raman spectroscopy as a diagnostic tool in the prediction, detection and evaluation of cancer tissues has been gaining considerable momentum in recent years. In particular, new techniques designed to enhance the intensity of Raman peaks, such as resonance Raman or surface enhanced Raman, and the attenuation or even elimination of fluorescence in biological samples by using near infrared incidence lasers, have highlighted the potential of using this type of spectroscopy in a clinical setting. The ability to correlate Raman spectra with specific locations within a sample, afford a cellular and even a subcellular glimpse into the molecular make-up of that given sample. In order to tap into this very promising potential, it would be imperative to determine in each case which molecular details would provide a unique signature that would allow a definitive discernment between healthy and cancerous tissues. In this context, it has been observed in recent years that various types of cancers exhibit an overexpression of aromatic amino acids. Since aromatic amino acids play important roles in a variety of metabolic processes, as well as key functions in the structural integrity of cellular and membrane proteins, anecdotal observations regarding their enhanced presence in cancerous tissues has given way to more dedicated and targeted inquiries. Given some observations that we have also made in our recent exploration of the use of SERS for the identification of breast cancer tissue, we have set out to probe the role of aromatic amino acids in breast cancer and to ascertain the possibility of using their overexpression as a diagnostic tool. The current work is a compilation of all pertinent publications dealing with the characterization of breast cancer cell lines and tissues using various modalities of Raman spectroscopy. All the Raman spectra in these papers, irrespective of the methodology used, have been normalized and have undergone detailed spectroscopic, chemical and statistical analysis. The emphasis of the analysis centered on the spectral region of 500–1200 cm^–1^ that has been shown to be the most characteristic for aromatic amino acids, specifically tryptophan, phenylalanine and tyrosine. We have been able to demonstrate that cancerous breast tissues and cells markedly exhibit overexpression of aromatic amino acids and that the difference between the extent of their presence in cancerous cells and healthy cells is decisive. The hypothesis for this work does not seek to establish Raman spectroscopy as a de-facto standard for the prediction or diagnosis of cancer, but rather as a tool with the ability to add, as well as complement, to the myriad of tools available to doctors and patients. Based on this analysis of the pertinent literature, we conclude that it may be possible to use the signature Raman bands of aromatic amino acids as a biomarker for the detection, evaluation and diagnosis of breast cancer, paving the way for future work by those in the field to set up in-vitro experiments and, hopefully, in-vivo work as well.

## Methods

The criteria for the consideration of the various papers for this study, and hence, the inclusion of the data that they contained were two-fold: (1) The papers had to focus on the characterization of breast tissue with Raman spectroscopy, and (2) the spectra provided within these papers included the spectral range of 500–1200 cm^−1^, which constitutes the characteristic region for aromatic amino acid vibrational modes. After all the papers that satisfied these criteria were collected, the relevant spectra from each paper were extracted. Each spectrum was then uploaded into Photoshop and a baseline was established. Once the baseline was determined, the intensities of the peaks corresponding to aromatic amino acids at 503, 657, 816, 837, 845, 965, 1006, 1012 and 1131 cm^−1^ were measured together with the highest intensity peak in the specific spectrum. Each peak was then normalized against the corresponding highest intensity band of the specific spectrum. Each such data point was then classified according to the type of breast tissue or breast cell line, i.e. healthy or cancerous, the frequency of the incident laser and the type of Raman technique.

It is important to note that the band processing protocol that we have used, while intending to minimize variance among the multitude of extracted spectra, nevertheless carries an associated possible error in the range of + /− 6 cm^−1^. This is due to several reasons: (1) The experimental resolution of the source Raman spectra, (2) The resolution of the reproduction of the spectra within the publication, and (3) The resolution of the digitization process and frequency assignment. The implication of this error range is that it could potentially cause an overlap between the Raman band of phenylalanine at 1006 cm^−1^ and the weak Raman band of tryptophan peak at 1012 cm^−1^, resulting in a more intense tryptophan Raman peak. However, since the Raman band of tryptophan peak at 1012 cm^−1^ is rather weak, this did neither pose a problem nor did it affect the analysis.

Of the several laser frequencies that have been used as the Raman excitation in the various papers that we have examined, we chose to concentrate on the results obtained with the 785 nm excitation laser. Raman spectra of biological samples tend to exhibit strong background due to tissue autofluorescence, and hence, can often lead to the attenuation of the weak Raman scattering signal. Among the several strategies to reduce tissue autofluorescence and improve the signal-to-noise ratio (SNR), the use of excitation lasers with wavelengths in the near-infrared range^50–53^ has proven effective. However, while longer excitation wavelengths effectively reduce tissue autofluorescence background, it also decreases the Raman signal intensity. Hence, the use of the 785 nm excitation laser provides an optimized balance between the reduction of background autofluorescence and band intensity.

## Data Availability

All data generated or analyzed during this study are included in this published article.
